# Shen Qi Li Xin formula improves chronic heart failure through balancing mitochondrial fission and fusion via upregulation of PGC-1α

**DOI:** 10.1186/s12576-021-00816-y

**Published:** 2021-10-18

**Authors:** Yan-Bo Sui, Jian Xiu, Jin-Xuan Wei, Pei-Pei Pan, Bi-Hong Sun, Li Liu

**Affiliations:** 1 Department of Cardiology, First Affiliated Hospital of Heilongjiang University of Chinese Medicine, No 26 Heping Road, Xiangfang District, Harbin, 150040 China; 2 Department of Cardiology, Heilongjiang University of Chinese Medicine, No 24 Heping Road, Xiangfang District, Harbin, 150040 China; 3 Department of Cardiology, First People’s Hospital of Zhaoqing, No 9 Donggangdong Road, Duanzhou District, Zhaoqing, China

**Keywords:** Chronic heart failure, Shen Qi Li Xin formula, Mitochondrial fission, Mitochondrial fusion, Cardiac function

## Abstract

**Background:**

Our previous study proved that Shen Qi Li Xin formula (SQLXF) improved the heart function of chronic heart failure (CHF) patients, while the action mechanism remains unclear.

**Methods:**

H&E staining and TUNEL staining were performed to measure myocardial damages. Western blot was used to examine the expression of proteins. Moreover, CCK-8 assay and flow cytometry were used to measure cell viability and cell apoptosis, respectively. Concentrations of ATP and ROS in cells, and mitochondrial membrane potential (MMP) were detected to estimate oxidative stress.

**Results:**

In vivo, we found that SQLXF improved cardiac hemodynamic parameters, reduced LDH, CK-MB and BNP production, and attenuated myocardial damages in CHF rats. Besides, SQLXF promoted mitochondrial fusion-related proteins expression and inhibited fission-related proteins expression in CHF rats and oxygen glucose deprivation/reoxygenation (OGD/R)-induced cardiac myocytes (CMs). In vitro, our data show that certain dose of SQLXF inhibited OGD/R-induced CMs apoptosis, cell viability decreasing and oxidative stress.

**Conclusion:**

Overall, certain dose of SQLXF could effectively improve the cardiac function of CHF rats through inhibition of CMs apoptosis via balancing mitochondrial fission and fusion. Our data proved a novel action mechanism of SQLXF in CHF improvement, and provided a reference for clinical.

**Supplementary Information:**

The online version contains supplementary material available at 10.1186/s12576-021-00816-y.

## Introduction

Chronic heart failure (CHF) is a common cardiovascular disease, with high morbidity rate, high mortality rate, high medical costs, and high hospital readmission [1]. CHF is a serious health problem globally. Rheumatic heart disease, hypertensive heart disease, chronic obstructive pulmonary disease and ischemic heart disease need to be responsible for more than 2/3 heart fail patients [2]. At present, medication still is the major therapeutic method of CHF, especially is in the CHF patients with reduced ejection fraction. The common drugs for CHF treatment include diuretics, inhibitors of angiotensin-converting, beta-blockers and other drugs [3]. Although a great development has been obtained in CHF treatment, there is still increasing in CHF morbidity and mortality.

Heart is the most main energy-consuming organ in human body. Mitochondrion is the major energy factory in cells. Mitochondrial homeostasis is a necessary condition for cellular physiological functions [4]. It was reported that mitochondrial dysfunction plays a crucial role in CHF occurrence and development, which is mainly embodied in the damage of mitochondrial DNA, high level of reactive oxygen species (ROS), increasing in mitochondrial membrane potential (MMP) and decreasing in ATP level [5, 6]. Under physiological condition, the fission and fusion of mitochondria always maintain balance, while the balance is broken in CHF. It was demonstrated that the expression of mitochondrial fission and fusion regulator, peroxisome proliferator-activated receptor γ co-activator 1 alpha (PGC-1α), is increased in CHF rat. Meanwhile, serious mitochondrial fission and attenuated fusion also were found in CHF [7, 8]. Mitofusin 1 (Mfn1), Mfn2 and optic atrophy 1 (Opa1) are three important regulators in fusion of mitochondria. Fission of mitochondria is mainly regulated by dynamin-related protein 1 (DRP1), fission 1 (Fis1) and mitochondrial fission factor [9].

Complementary medicine, especially traditional Chinese medicine (TCM), has more advantages in the treatment of many types of disorders, such as in CHF [10]. Zhao et al. reported that TCM Qiliqiangxin capsule could effectively protect SD rats against CHF via inhibiting mitochondrial fission and attenuating oxidative stress-induced apoptosis in cardio myocytes (CMs) [11]. In clinical trial, we found that Shen Qi Li Xin formula (SQLXF), one of TCM composed of Ginseng, Astragalus, Semen lepidii and other herb-medicines, effectively improve the ventricular function in patients with CHF [12]. In this present study, we further explore the action mechanism of SQLXF in CHF improvement in animal model and cell model. Here, we revealed that SQLXF protected ventricular function in CHF rats through inhibition of CMs apoptosis via balancing PGC-1α-mediated mitochondrial fission and fusion. Our data proved a novel evidence to support SQLXF as CHF treatment drugs, and supplied a new idea for CHF treatment.

## Materials and methods

### Animal model and experimental groups

A total of 30 Sprague–Dawley (SD) rats (male, 200 ± 20 g) were purchased from Charles River (Beijing, China). All rats live in a suitable environment with 12 h of light–dark cycles, enough water and enough food. All experiments in rats were approved by the Ethics Committee of the Ethics of Animal Experiments of Heilongjiang University of Chinese Medicine. All experiments were performed strictly in accordance with the requirement of the Guidelines for the Care and Use of Laboratory Animals of the Ministry of Science and Technology of China. All possible steps were taken to avoid animal suffering at each stage of the experiment.

All rats were anesthetized using 0.3% pentobarbital sodium at a dose of 10 ml/kg. Then, pericardium was opened to expose rats’ epicardium, the left anterior descending coronary artery of SD rats was ligated using a single 6–0 nylon suture between auricular appendix and conus arteriosus to establish CHF rats. The ligated rats with left ventricular ejection fraction ≤ 45% was recognized as a successful CHF rat model. All rats were randomly divided into five groups (*n* = 6): sham, model, low, middle and high. In sham group, all rats underwent the same procedure, but without ligation. In low group,  the CHF rats were treated with SQLXF at a dose of 8.48 g/kg/day through intragastric administration. In middle group, the CHF rats were treated with SQLXF at a dose of 16.96 g/kg/day through intragastric administration. In high group, the CHF rats were treated with SQLXF at a dose of 33.92 g/kg/day through intragastric administration. In the sham group and the model group, the rats were treated with saline through intragastric administration. The second day after operation, CHF rats began to receive SQLXF treatment. SQLXF: Ginseng 15 g, Astragalus 30 g, Semen lepidii 15 g, Poria 15 g, Salvia 15 g, Cortex moutan 15 g, Ramulus cinnamomi 10 g.

At 4 weeks after SQLXF treatment, serum samples were obtained from each rat. Next, the concentration of lactate dehydrogenase (LDH) and creatine kinase-MB (CK-MB) in serum were analyzed using a LDH assay kit (Abcam, Cambridge, England, U.K.) and CK-MB assay kit (Wuhan Easydiagnosis Biomedicine Co., Ltd, Wuhan, China), respectively. Besides, serum brain natriuretic peptide (BNP) level was analyzed using a BNP ELISA kit (Abcam). All detections were accomplished in accordance with the specific manufacture’s introduction.

### Evaluation of cardiac hemodynamic parameters

After conduction echocardiography, all rats were anesthetized with 20% urethane at a dose of 5 ml/kg. All rats were then fixed on an operation table. Next, a micro-catheter, linked with a pressure transducer, was inserted into the left ventricular from right common carotid artery. Subsequently, left ventricular systolic pressure (LVSP), left ventricular end-diastolic pressure (LVEDP), maximal positive rate of developed left ventricular pressure (+LVdP/dtmax) and maximal negative rate of developed left ventricular pressure (−LVdP/dtmax) in rats were recorded using a multichannel physiological recorder. +LVdP/dtmax is an indicator of myocardial contraction. −LVdP/dtmax is a meter of myocardial relaxation.

### H&E staining

At 4 weeks after SQLXF treatment, myocardial tissues were obtained from all rats. Next, paraffin-embedded myocardial tissues were cut into serial section with 4 μm thick. Subsequently, sections were stained with H&E (Nanjing Jiancheng Bioengineering Institute, Nanjing, China) according to the kit protocol. The pathological changes in rats were observed using a microscope (Olympus Medical Systems Corp, Tokyo, Japan) and analyzed.

### TUNEL staining

Cell apoptosis in myocardial tissues of rats was measured by TUNEL staining. After routinely dewaxing and hydration, sections of myocardial tissues were stained with TUNEL reagent (Solarbio, Beijing, China) for 30 min at 37 °C in the dark. DAPI was utilized to stain nucleus for 5 min. At last, the apoptotic cells were observed under a confocal laser scanning microscope. The number of TUNEL-positive cells was analyzed.

### Western blot assay

Total protein was isolated from myocardial tissues and H9c2 cell using RIPA lysis buffer (Santa Cruz Biotechnology, Texas, USA). After detection of the concentration of proteins, an equal amounts of 20 μg protein was mixed with 5 × loading buffer. Then, the mixture was separated on a 12% SDS-PAGE gel, and then was transferred onto a PVDF membrane (Boster, Wuhan, China). After that, the membranes were maintained with 5% non-fat milk for 1 h at room temperature. Subsequently, the membranes were incubated with antibodies working solution of PGC-1α (1:2000, ab106814, Abcam), Mfn2 (1:2000, ab124773, Abcam), Opa1 (1:2000, ab42364, Abcam), Drp1 (1:2000, ab184247, Abcam) and Fis1 (1:2000, ab96764, Abcam) at 4 °C overnight. Next day, the membranes were maintained with secondary antibodies for 1 h at room temperature. At last, the membranes were maintained with ECL reagent (Beyotime Biotechnology, Haimen, China) to display protein bands. The relative expressions of proteins were normalized to β-actin.

### Preparation of medicated serum

In TCM-related experiments, medicated serum was made usually used to carry out celluer experiments, that is due to most TCM is oral medicine, medicated serum can better reflect the efficacy of TCM, medicated serum is easy to preserve and other factors [13, 14]. Another 40 SD rats were purchased for preparation of medicated serum. All rats were randomly divided into four groups (*n* = 10): blank, low-dose, middle-dose and high-dose. In low-dose, middle-dose and high-dose groups, the rats were fed with Chinese herbal decoction of SQLXF at the doses of 8.48, 16.96 and 33.92 g/kg, respectively, once a day for 7 days. In blank group, the rats were fed with equal volume of normal saline, once a day for 7 days. Next, after 2 h of the last gavage, abdominal aortic blood was collected under sterile conditions. Then, the blood samples were centrifuged at 3000 r/min for 15 min to obtain serum. After filtering through a 0.22-μm microporous membrane, the serum was inactivated at 56 °C for 30 min and stored at − 20 °C.

### Cell culture and experimental groups

CM H9c2 (rat embryonic ventricular myocytes) was from National Science & Technology Infrastructure (Shanghai, China). In our study, all cells were cultured in Dulbecco’s modified Eagle’s medium (DMEM; Invitrogen, Carlsbad, CA, USA) supplemented with 10% fetal bovine serum (FBS; Invitrogen) and 1% antibiotics (Invitrogen). Cells were grown at 37 °C in an incubator with 5% CO_2_. Cells were divided into six groups: Ctrl, oxygen glucose deprivation/reoxygenation (OGD/R), OGD/R + blank serum, OGD/R + low-dose serum, OGD/R + middle-dose serum and OGD/R + high-dose serum. In OGD/R + blank serum group, cells were given the serum (10 μl) from the rats in blank group. In OGD/R + low-dose serum group, cells were treated with the serum (10 μl) from the rats in low-dose group. In OGD/R + middle-dose serum group, cells were given the serum (10 μl) from the rats in middle-dose group. In OGD/R + high-dose serum group, cells were given the serum (10 μl) from the rats in high-dose group. The cells in Ctrl and OGD/R groups were treated with PBS instead of medicated serum.

H9c2 cells were grown in normal conditions for 48 h. For OGD/R experiments, the cells were cultured in glucose-free DMEM medium (Gibco, Cat. No. 11966025) supplemented with 1% FBS, and were exposed to hypoxic conditions (1% O_2_) for 24 h at 37 °C in a three-gas incubator (Thermo Fisher scientific, USA). Then, the cells were cultured in normal conditions again for another 24 h.

### CCK-8 assay

CCK-8 assay was carried out to detect cell viability. H9c2 cells were seeded in into 96-well plates at a density of 1 × 10^4^ per well, and were treated with medicated serum containing different SQLXF doses. After 24 h of reoxygenation, 10 μl of CCK-8 solution (MedChemExpress, USA) was added into each well. Cells were incubated with CCK-8 solution for another 2 h. Next, the absorbance of solution at 450 nm was measured using a Synergy H1 Hybrid Reader (Biotech, USA).

### Flow cytometry assay

Cell apoptosis was measured by flow cytometry. H9c2 cells were planted into 6-well plates at a density of 1.5 × 10^6^ per well. Then, the cells were given medicated serum and OGD/R stimulation. After 24 h of reoxygenation, the cells were fixed with 4% paraformaldehyde. Subsequently, a FITC Annexin V Apoptosis Detection Kit (BD Pharmingen™, USA) was utilized to detect the rate of apoptotic cells in accordance with the manufacturer’s protocol.

### Detection of ATP, ROS and MMP in CMs

At 48 h after SQLXF treatment, the concentration of ATP in CMs was measured by bioluminescence assay using an ATP assay kit (Solarbio). Concentration of ROS in CMs was examined using a Reactive Oxygen Species Assay Kit (Solarbio). Mitochondrial Membrane Potential Assay Kit with JC-1 was used to measure MMP of CMs. All experiments were carried out according to the kits’ protocol. In accordance with the levels of ATP, ROS and MMP were measured to assessm oxidative stress and mitochondrial damage.

### Statistical analysis

SPSS 20.0 software (SPSS Inc., Chicago, IL, USA) was utilized to analyze all data difference. Data were presented as mean ± standard deviation (SD). Comparison between two independent groups was determined by Student’s *t*-test. One-way ANOVA was used to analyze the significant difference among three groups. *P* < 0.05 was considered statistically significant.

## Results

### SQLXF effectively attenuated myocardial damage in CHF rats

**Fig. 1:**
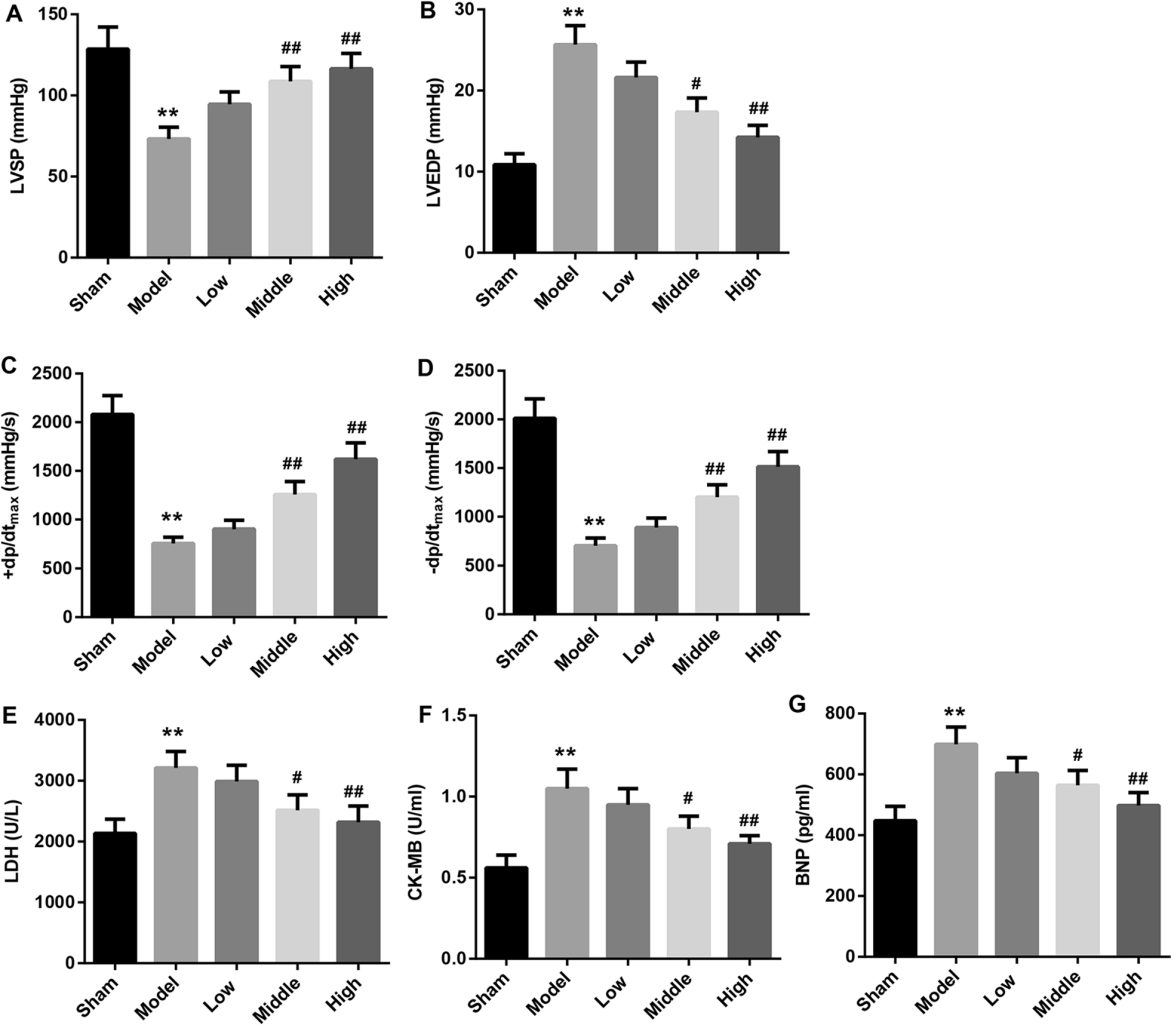
Effect of SQLXF on the left ventricular functions of CHF rats. CHF rats were treated with low-dose (8.48 g/kg/day), middle-dose (16.96 g/kg/day), and high-dose (33.92 g/kg/day) SQLXF, respectively. **A** LVSP in all rats was measured. **B** LVEDP in all rats was examined. **C** + LVdP/dtmax in all rats was measured. **D** −LVdP/dtmax in all rats was measured. **E** Concentration of LDH in the serum of all rats was measured using LDH detection kit. **F** Concentration of CK-MB in the serum of all rats was examined using a specific kit. **G** ELISA assay was carried out to detect the concentration of BNP in the serum of all rats. **P < 0.01 compared with Sham group. ^#^*P* < 0.05 and ^##^*P* < 0.01 compared with Model group

**Fig. 2:**
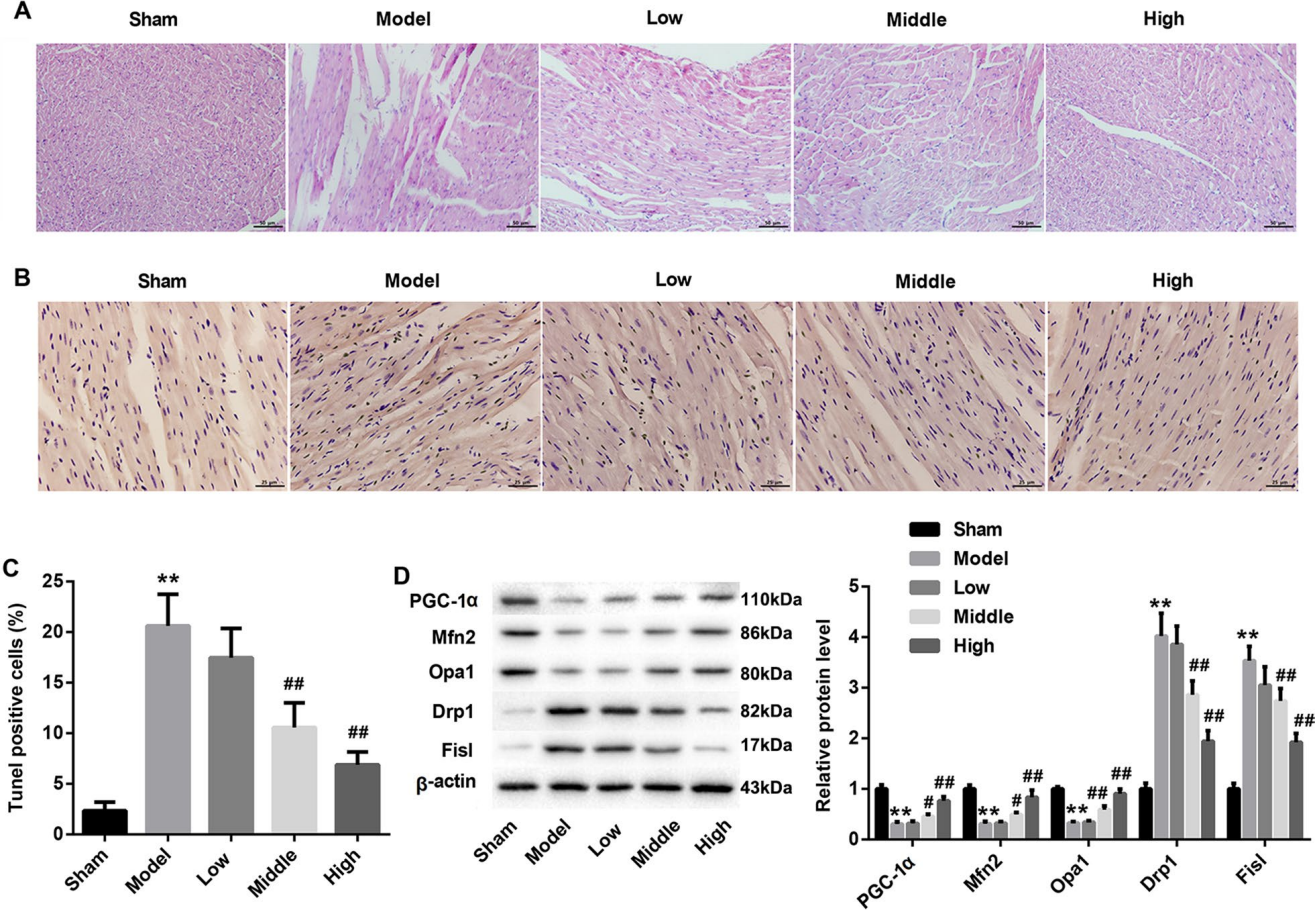
Effect of SQLXF on the myocardial injury in CHF rats. **A** H&E staining was performed to detect the pathological changes in myocardial tissues of rats. **B** TUNEL experiments were fulfilled to detect cell apoptosis in myocardial tissues of rats. **C** Rate of apoptotic cells in myocardial tissues of rats was analyzed. **D** Western blot was performed to measure the expression of PGC-1α, Opa1, Mfn2, Drp1 and Fis1 in myocardial tissues of rats. **P < 0.01 compared with Sham group. ^#^*P* < 0.05 and ^##^*P* < 0.01 compared with Model group

CHF rats were treated with SQLXF at a doses of 8.48, 16.96 or 33.92 g/kg/day. To investigate the effect of SQLXF on the ventricular functions of CHF rats, we detected the levels of hemodynamic parameters, including LVSP, LVEDP, + LVdP/dtmax and −LVdP/dtmax. As shown in Fig. 1A–D, our data proved that the levels of LVSP, + LVdP/dtmax and −LVdP/dtmax in CHF rats were downregulated, and LVEDP level in CHF rats was upregulated. However, 16.96 and 33.92 g/kg/day doses of SQLXF could notably increase the levels of LVSP, + LVdP/dtmax and -LVdP/dtmax, and reduce the level of LVEDP. Moreover, our data also indicated that the levels of serum LDH (Fig. 1E), CK-MB (Fig. 1F) and BNP (Fig. 1G) were significantly upregulated in CHF rats. However, 16.96 and 33.92 g/kg/day doses of SQLXF treatment could effectively downregulate the levels of serum LDH, CK-MB and BNP in CHF rats. Furthermore, seriously myocardial tissue damage was found in CHF rats compared with normal rats. The myocardial tissues of normal rat exhibit tightly arranged myocardial fibers, and without obvious deformation, edema and inflammatory cell infiltration. The myocardial tissues of CHF rat model exhibit broken and necrotic in myocardial fibers, and a large number of inflammatory cells infiltration into interstitial, while SQLXF treatment at a doses of 16.96 and 33.92 g/kg/day could obviously attenuate the damage in CHF rats (Fig. 2A). Furthermore, our results showed that the numbers of apoptotic cell in myocardial tissue from CHF rats higher than that from normal rats. SQLXF treatment could obviously suppress the cell apoptosis in myocardial tissues from CHF rats (Fig. 2B and 2C). The expression of cleaved caspase-3, an apoptosis-related protein, was increased in the myocardial tissues of CHF rats compared to normal rats, which was downregulated by SQLXF treatment (Additional file 1: Figure S1A). Next step, our data demonstrated that the expression of PGC-1α in myocardial tissues of CHF rats was inhibited. The expression of mitochondrial fusion proteins, Mfn2 and Opa1, were also downregulated in myocardial tissues of CHF rats, and mitochondrial fission proteins, Drp1 and Fis1, were downregulated. Importantly, 16.96 and 33.92 g/kg/day doses of SQLXF significantly promoted the expression of PGC-1α, Mfn2 and Opa1, inhibited Drp1 and Fis1 expression in myocardial tissue from CHF rats (Fig. 2D). Overall, SQLXF at a doses of 16.96 and 33.92 g/kg/day effectively balanced PGC-1α-mediated fission and fusion of mitochondria, and improved damage in myocardial tissue of CHF rats.

### SQLXF suppressed cell apoptosis and balanced mitochondrial fusion and fission in OGD/R-induced H9c2 cells

**Fig. 3:**
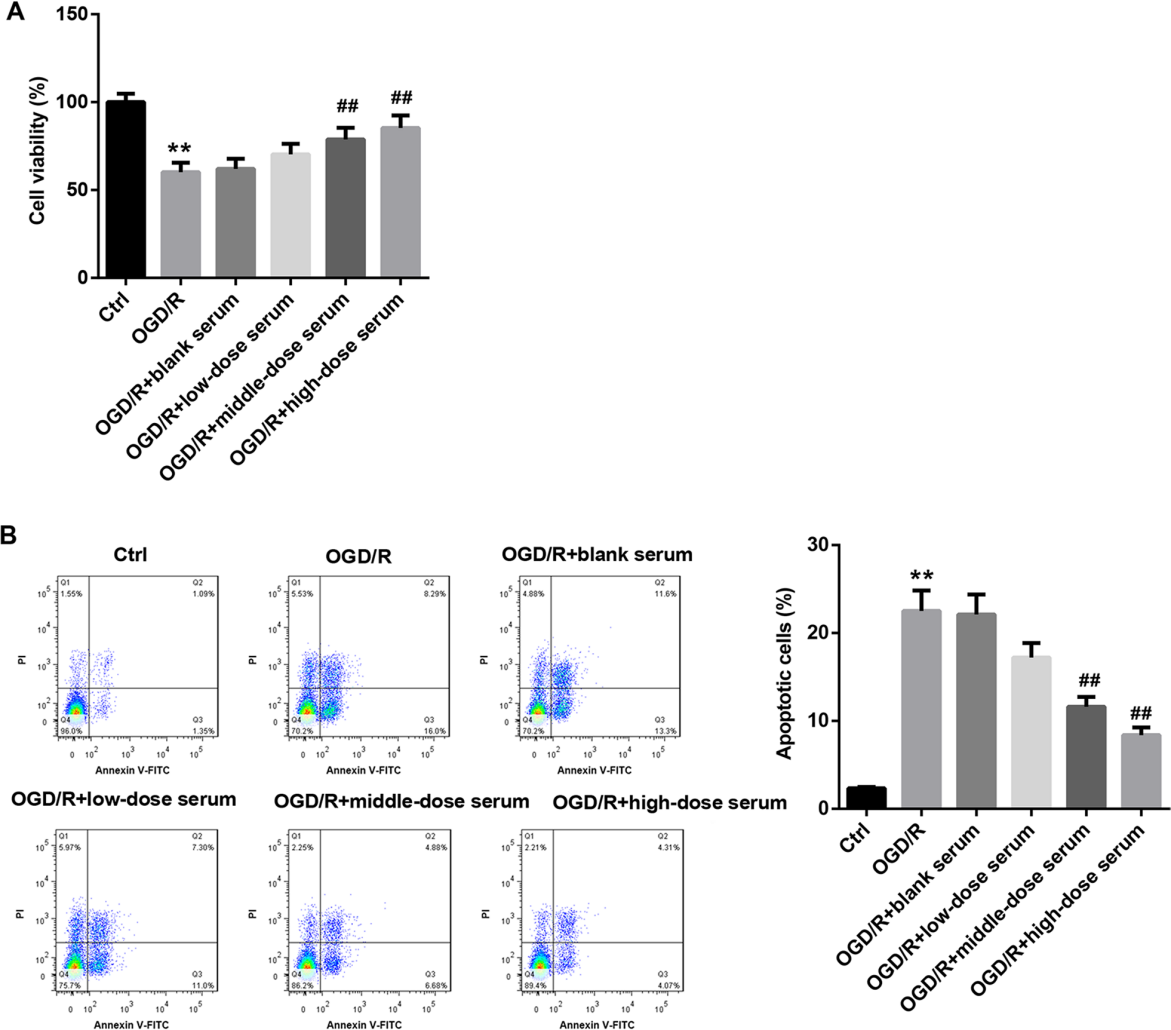
Effect of SQLXF on viability and apoptosis of OGD/R-stimulated H9c2 cells. H9c2 cells were incubated with rat’s medicated serum containing SQLXF at different doses. OGD/R was used to induce H9c2 cells injury. **A** CCK-8 was carried out to examine cell viability of H9c2 cells. **B** Flow cytometry was performed to measure cell apoptosis of H9c2 cells. **P < 0.01 compared with Ctrl group. ^##^*P* < 0.01 compared with OGD/R group

**Fig. 4:**
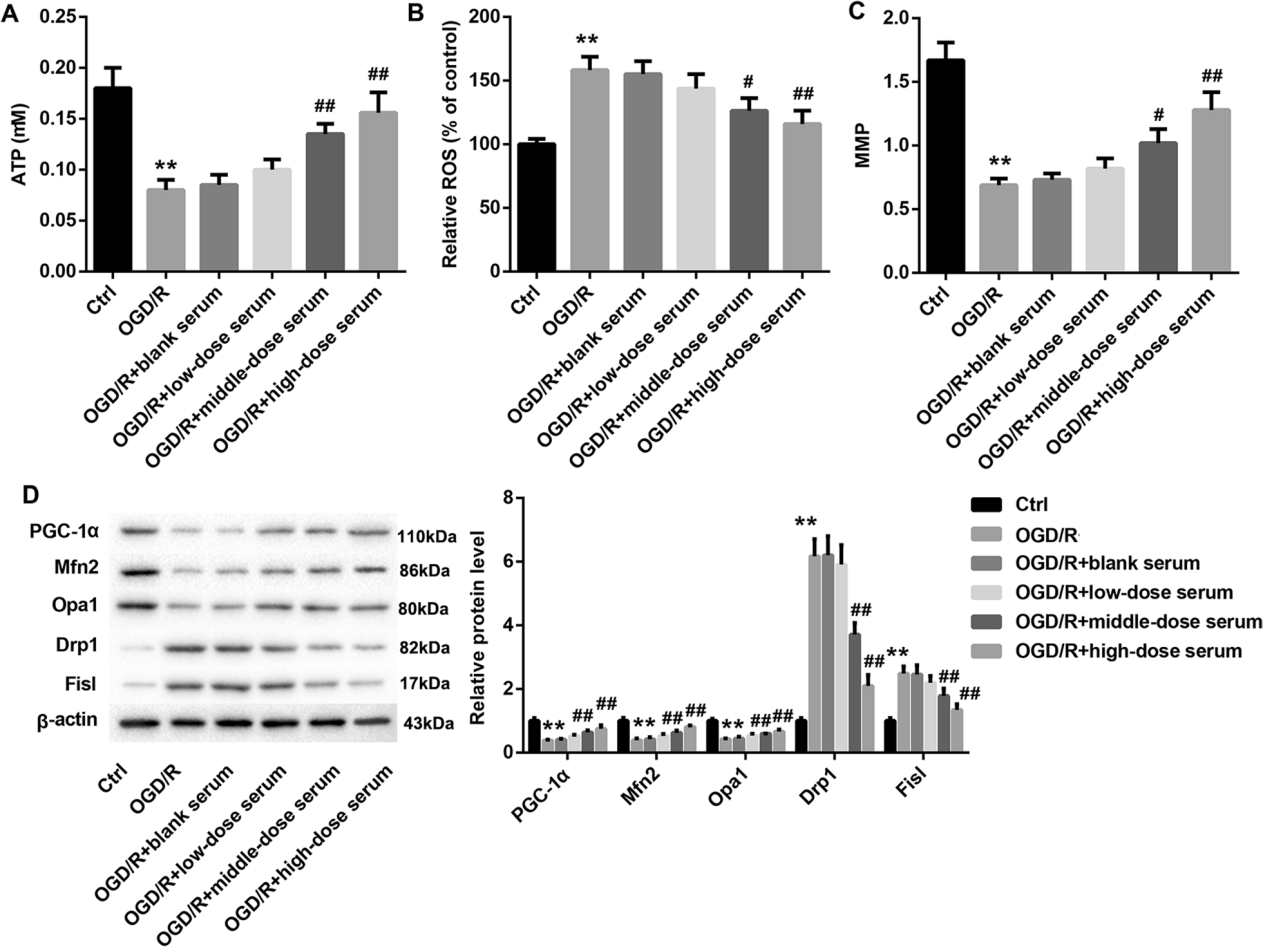
Effect of SQLXF on mitochondrial fusion and fission in OGD/R-stimulated H9c2 cells. **A** Intracellular ATP content in H9c2 cells was examined by bioluminescence assay. **B** Mitochondrial ROS generation was qualitatively observed using a DCFH-DA kit. **C** Fluorescent probe of JC-1 was used to measure MMP in H9c2 cells. **D** Expression of PGC-1α, Mfn2, Opa1, Drp1 and Fis1 was examined using Western blot assay. **P < 0.01 compared with Ctrl group. ^#^*P* < 0.05 and ^##^*P* < 0.01 compared with OGD/R group

Subsequently, the rats medicated serum containing SQLXF at a doses of 8.48, 16.96 or 33.92 g/kg was made, which was then incubated with OGD/R-treated H9c2 cells. Our data indicated that OGD/R markedly downregulated the cell viability, and the medicated serum containing SQLXF at a doses of 16.96 and 33.92 g/kg enhanced the cell viability of OGD/R-induced H9c2 cells (Fig. 3A). Meanwhile, OGD/R caused a high level of cell apoptosis in H9c2 cells and promoted the expression of cleaved caspase-3. Medicated serum containing SQLXF at a doses of 16.96 or 33.92 g/kg effectively suppressed OGD/R-induced cell apoptosis and downregulated cleaved caspase-3 expression (Fig. 3B and Additional file 1: Figure S1B). In addition, our data further proved that the production of ATP was reduced in OGD/R-stimulated H9c2 cells. Medicated serum containing SQLXF at a doses of 16.96 and 33.92 g/kg treatment enhanced the production in OGD/R-treated H9c2 cells (Fig. 4A). Oppositely, the production of ROS in H9c2 cells was promoted by OGD/R induction, while medicated serum containing SQLXF at a doses of 16.96 and 33.92 g/kg treatment could obviously reduce the production of ROS (Fig. 4B). OGD/R treatment also downregulated MMP expression in H9c2 cells, and medicated serum containing SQLXF at a doses of 16.96 and 33.92 g/kg treatment boosted MMP production (Fig. 4C). Importantly, our data showed that the expression of PGC-1α, Mfn2 and Opa1 were downregulated in OGD/R-treated H9c2 cells, and Drp1 and Fis1 expression were upregulated in the cells. However, medicated serum containing SQLXF at doses of 16.96 and 33.92 g/kg treatment could effectively facilitate PGC-1α, Mfn2 and Opa1 expression, and impede Drp1 and Fis1 expression (Fig. 4D). In summary, SQLXF notably inhibited OGD/R-induced CMs apoptosis through regulation the balance of mitochondrial fusion and fission.

## Discussion

Prolonged uses of chemical drugs may result in serious side effects on CHF patients, for example, hypotension and electrolyte depletion. TCM has been considered as an alternative method for CHF treatment [15]. In China, TCM was utilized to great majority disorders therapy. Compared with chemical drugs, TCM has better therapeutic effect and lesser side effects in many disorders [16, 17]. SQLXF is Chinese herbal medicine compound preparation, composed of radix astragali, radix ginseng, monkshood, radix salviae miltiorrhizae and other herbal medicines. Clinical trials and basis experiments demonstrated that SQLXF effectively improves myocardial injury and cardiac function of the patients with CHF and CHF animal model [18].

In our previous study, we demonstrated that the cardiac output, cardiac index, left ventricular ejection fraction, left ventricular end-diastolic diameter and every cardiac output in CHF patients are significantly improved by SQLXF treatment [12]. Our previous study proved the improvement effect of SQLXF on cardiac function in the patients with CHF. Here, our data indicated that SQLXF at a dose of higher than 16.96 g/kg/day effectively improves the hemodynamic parameters and myocardial damage in CHF rats. Moreover, our results also revealed that SQLXF promotes mitochondrial fusion-related proteins expression, and inhibits mitochondrial fission-related proteins expression. SQLXF impedes OGD/R-induced mitochondrial oxidative stress, CMs apoptosis, and enhances cell viability in vitro.

Mitochondria dysfunction is a key feature of CMs dysfunction in CHF, and maybe a promising therapeutic target for the disease [19]. Mitochondria are the major cellular energy-producing organelles and intracellular source of ROS, and it also as playmakers of apoptosis, autophagy and senescence [20, 21]. Normal fusion and fission in mitochondrial and mitophagy are an important factor for the maintain of mitochondrial quality [22]. It was revealed that imbalanced mitochondrial fission and fusion contributes to heart failure and other cardiovascular diseases development. A drug that can be used to balance the inclined mitochondrial fission and fusion may be a hope for CHF treatment [23]. PGC-1α is a transcription factor with 91 kDa quality, and is a co-activator in mitochondrial biogenesis. Knockout of PGC-1α in mouse could result in the reduction in ATP level and mitochondrial enzymatic activities, finally leading to heart failure [24]. In this study, our results indicated that PGC-1α is downregulated in both CHF rats and OGD/R-stimulated CMs. SQLXF treatment could effectively facilitate the expression of PGC-1α. Mfn2 and Opa1 are two crucial regulators in mitochondrial fusion. It was reported that Mfn2 expression will be downregulated after PGC-1α depletion [25]. Here, our data showed that the expression of Mfn2 and Opa1 in CHF rats and OGD/R-induced CMs were inhibited, while SQLXF treatment could obviously upregulate their expression. Furthermore, Drp1 and Fis1 are two crucial regulators in mitochondrial fission. Mitochondrial fission factor and Fis1 facilitate the transfer of Drp1 to mitochondria, and then contribute the fission in mitochondria [26]. In the present study, our results suggested that Drp1 and Fis1 are increased in CHF rats and OGD/R-stimulated CMs, but SQLXF treatment effectively suppresses their expression. Recently, some studies revealed that mitochondrial dysfunction participates in the CMs apoptosis under certain pathological conditions [27]. For instance, melatonin could protect against lipopolysaccharide-induced CMs autophagy and apoptosis through facilitating mitochondrial uncoupling protein 2 expression and mitochondrial homeostasis [28]. Nevertheless, it is unclear that whether SQLXF improves the CMs apoptosis in CHF through mitochondria. On one hand, our results are consistent with previous studies. In CHF, cardiac function is damaged, cell apoptosis is increased, mitochondrial fusion is inhibited, and mitochondrial fission is facilitated in heart tissues [29, 30]. On the other hand, our study showed some new results. SQLXF effectively improves the cardiac function of CHF rats and balances mitochondrial fission and fusion.

## Conclusion

In conclusion, our data demonstrated that certain dose of SQLXF could effectively protect cardiac function in CHF rats through suppressing CMs apoptosis via balancing the fission and fusion of mitochondria. Our data prove a novel regulatory mechanism for SQLXF improve CHF.

### Supplementary Information

**Additional file 1: Figure S1.** Effect of SQLXF on the apoptosis of OGD/R-treated H9c2 cells. (A) CHF rats were treated with low-dose (8.48 g/kg/d), middle-dose (16.96 g/kg/d), and high-dose (33.92 g/kg/d) SQLXF, respectively. The expression of cleaved caspase-3 in myocardial tissues was measured using Western blot. (B) H9c2 cells were incubated with rat’s medicated serum containing SQLXF at different doses. The expression of cleaved caspase-3 in H9c2 cells was measured using Western blot.

## Data Availability

The datasets used and/or analyzed during the current study are available from the corresponding author on reasonable request.
