# Correction to: Shen Qi Li Xin formula improves chronic heart failure through balancing mitochondrial fission and fusion via upregulation of PGC-1α

**DOI:** 10.1186/s12576-022-00840-6

**Published:** 2022-10-03

**Authors:** Yan-Bo Sui, Jian Xiu, Jin-Xuan Wei, Pei-Pei Pan, Bi-Hong Sun, Li Liu

**Affiliations:** 1 Department of Cardiology, First Affiliated Hospital of Heilongjiang University of Chinese Medicine, No 26 Heping Road, Xiangfang District, Harbin, 150040 China; 2 Department of Cardiology, Heilongjiang University of Chinese Medicine, No 24 Heping Road, Xiangfang District, Harbin, 150040 China; 3 Department of Cardiology, First People’s Hospital of Zhaoqing, No 9 Donggangdong Road, Duanzhou District, Zhaoqing, China

## Correction to: J Physiol Sci (2021) 71:32 https://doi.org/10.1186/s12576-021-00816-y

In this article, the author would like to add the funding information as “the Open fund of Key Laboratory of Ministry of Education for TCM Viscera-State Theory and Applications, Liaoning University of Traditional Chinese Medicine.”

The original article [1] has been corrected.
