# Supplementary figures and images for: Shen Qi Li Xin formula improves chronic heart failure through balancing mitochondrial fission and fusion via upregulation of PGC-1α
Source: J Physiol Sci. 2021 Oct 18;71:32. doi: 10.1186/s12576-021-00816-y (PMC10717454; doi:10.1186/s12576-021-00816-y)

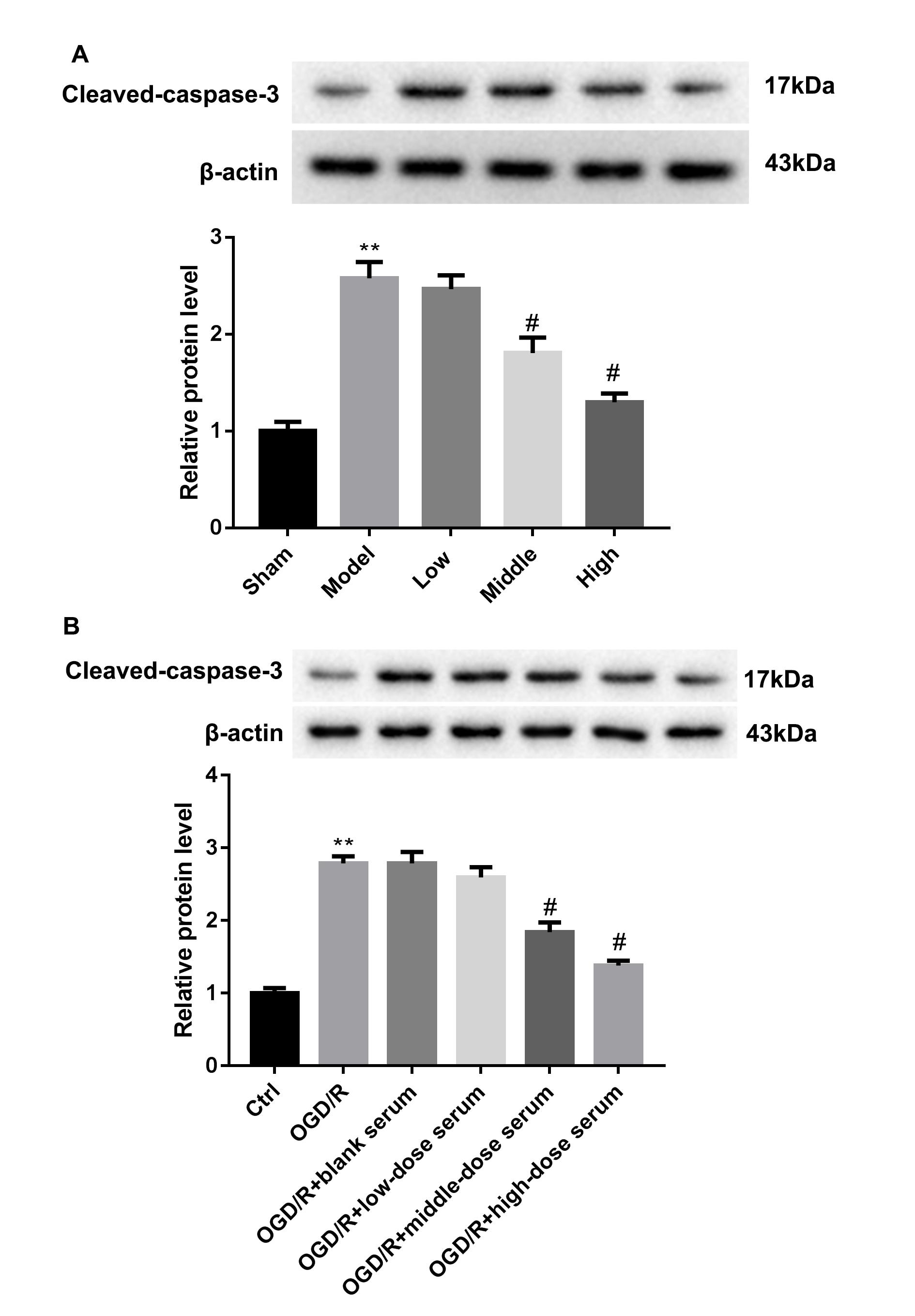

Supplement: Supplementary file 1 — Additional file 1: Figure S1. Effect of SQLXF on the apoptosis of OGD/R-treated H9c2 cells. (A) CHF rats were treated with low-dose (8.48 g/kg/d), middle-dose (16.96 g/kg/d), and high-dose (33.92 g/kg/d) SQLXF, respectively. The expression of cleaved caspase-3 in myocardial tissues was measured using Western blot. (B) H9c2 cells were incubated with rat’s medicated serum containing SQLXF at different doses. The expression of cleaved caspase-3 in H9c2 cells was measured using Western blot. [file 12576_2021_816_MOESM1_ESM.tif]
